# Sequential Double Autotransplantation Using Immature Teeth as First‐Stage Autotransplants to Preserve Alveolar Architecture: A Case Series

**DOI:** 10.1155/crid/6280261

**Published:** 2026-06-20

**Authors:** Miks Lejnieks, Juan E. Onetto, Marie-Therese Flores, Andris Abeltins, Girts Salms, Sergio E. Uribe

**Affiliations:** ^1^ Department of Oral and Maxillofacial Surgery and Oral Medicine, Rīga Stradiņš University, Rīga, Latvia, rsu.lv; ^2^ Institute of Stomatology, Rīga Stradiņš University, Rīga, Latvia, rsu.lv; ^3^ Baltic Biomaterials Centre of Excellence, Headquarters at Rīga Technical University, Rīga, Latvia; ^4^ Department of General Dentistry, Rīga Stradiņš University, Rīga, Latvia, rsu.lv; ^5^ Department of Pediatric Dentistry, Faculty of Dentistry, Universidad de Valparaíso, Valparaíso, Chile, uv.cl; ^6^ Research Center in Dental and Medical Sciences (CICOM), Universidad de Valparaíso, Valparaiso, Chile, uv.cl; ^7^ Department of Orthodontics, Rīga Stradiņš University, Rīga, Latvia, rsu.lv

**Keywords:** autotransplantation, case report, immature tooth, pulp vitality, root development

## Abstract

**Introduction:**

Missing teeth in growing patients require treatment approaches that preserve alveolar bone and support continued development. Autotransplantation offers a biological alternative to implants, yet severely compromised recipient sites and immature donor teeth present clinical challenges. This case series describes a sequential double autotransplantation protocol using immature first‐stage autotransplants to preserve alveolar architecture and exploit the osteogenic potential of periodontal ligament‐derived mesenchymal stromal cells for alveolar bone regeneration.

**Methods:**

Three patients (ages 16–18 years) with severe bone defects, oroantral communication, or cystic pathology underwent planned two‐stage autotransplantation. CBCT‐guided planning and 3D‐printed templates were used to minimize extra‐alveolar time. Immature donor teeth were used as first‐stage transplants to preserve alveolar architecture and periodontal space prior to definitive transplantation. Secondary transplantation with a more developmentally mature donor followed assessment at 12 months.

**Results:**

All cases achieved favorable outcomes at 24 months after second‐stage autotransplantation, including pulp chamber obliteration without periapical pathology or clinical symptoms, consistent with maintained pulp vitality, continued root development, and periodontal probing depths of 2–3 mm at final follow‐up.

**Conclusions:**

The primary lesson is that recipient site condition, rather than donor tooth development alone, determines prognosis in severely compromised cases, and a staged protocol can preserve alveolar architecture for definitive rehabilitation. Recipient site condition, rather than donor tooth developmental stage alone, appears to be the primary determinant of outcome in severely compromised cases. Validation through larger controlled studies remains essential.

## 1. Introduction

In growing patients, replacement of missing teeth must account for ongoing alveolar bone growth and root development. Preservation of the periodontal ligament is essential to maintain physiological bone remodeling and prevent complications such as ankylosis and root resorption [1]. Although implant therapy is effective in adults, it is contraindicated in growing patients due to continued skeletal development and the risk of infraocclusion and bone loss. Tooth autotransplantation preserves periodontal ligament function and supports alveolar growth in growing patients [2]. First reported by Nordenram [3] in 1963, autotransplantation has been shown to improve outcomes, particularly with respect to pulp healing and root development [4]. Long‐term outcomes demonstrate survival rates of 80%–97.5% with appropriate case selection, particularly for immature teeth [4]. CBCT planning with virtually planned 3D‐printed surgical templates enhances outcomes by reducing surgical time, improving precision, and minimizing Hertwig epithelial root sheath injury [5–7]. Root development stage remains critical for success [4, 8], with optimal timing occurring at specific developmental stages [9]. However, donor teeth with minimal development present compromised revascularization potential and suboptimal prognosis [10]. Clinical observations indicate that very immature donors often lack primary stability and continued root development, prompting development of a sequential protocol with planned replacement. Immature first‐stage autotransplants may help preserve alveolar architecture during healing. The regenerative potential of periodontal ligament‐derived cells has been described in previous studies [11] In cases with severely compromised recipient sites, conventional single stage autotransplantation demonstrated suboptimal outcomes, attributable primarily to recipient site biology rather than donor tooth characteristics. The staged protocol was therefore developed to optimize alveolar conditions prior to definitive transplantation: The first‐stage autotransplant was planned from the outset to preserve periodontal space and stimulate bone regeneration, ensuring sufficient alveolar architecture for the second‐stage definitive transplant and, if needed, for implant‐based rehabilitation once skeletal growth was complete. This approach was specifically designed for young growing patients in whom preserving future rehabilitation options is a critical treatment goal. To our knowledge, no previous case series has described this staged double autotransplantation approach in growing patients with severely compromised recipient sites. This case series presents three patients managed by sequential double autotransplantation. The sequential approach supported alveolar healing and mechanical stability through staged timing.

## 2. Materials and Methods

This case series was prepared in accordance with the CARE 2013 guidelines [12]. Patient consent for publication was obtained in all cases. Each case required specific modifications: management of an oroantral communication (Case 1), treatment of cystic pathology (Case 2), and concurrent orthodontic repositioning (Case 3). This case report series was conducted in accordance with the Declaration of Helsinki and institutional ethical guidelines. Ethical approval was obtained from the Rīga Stradiņš University Research Ethics Committee (No. 6‐1/08/12, dated July 23, 2020; 2_PĒK‐4/530/2026, dated April 16, 2026). Written informed consent for publication was obtained from all patients prior to manuscript preparation. Prior to the first surgical procedure, all patients were explicitly informed that the staged protocol involved a planned second autotransplantation. Informed consent covered both surgical stages.

### 2.1. Surgical Protocol

A common surgical approach was used, with case‐specific modifications based on anatomy and pathology [13]. All patients received preoperative antibiotic prophylaxis consisting of amoxicillin/clavulanic acid 875/125 mg (two tablets, maximum 20 mg/kg body weight), or clindamycin 600 mg (maximum 15 mg/kg) in cases of penicillin allergy, according to our previously published protocol [6]. In cases involving deeply impacted donor teeth, the standard postoperative regimen for impacted tooth surgery was applied: amoxicillin 500 mg twice daily for 7 days. Local anesthesia was achieved using 4% articaine with 1:100,000 epinephrine by infiltration. Donor teeth were extracted atraumatically with an osteotomy using straight fissure and surgical round burs, with saline irrigation and a maximum speed of 800 rpm. Extra‐alveolar time was minimized to preserve periodontal ligament viability. The recipient site was prepared by sequential osteotomy using straight fissure and surgical round burs, followed by Straumann BLT implant burs (Basel, Switzerland), with saline cooling via a KaVo Physio dispenser (KaVo, Biberach an der Riss, Germany) at a maximum speed of 800 rpm, ensuring precise dimensional matching to the donor root morphology [6]. Donor teeth were positioned in infraocclusion and stabilized with 4‐0 Vicryl sutures. Ibuprofen 400 mg every 8 h was prescribed as needed. Patients used chlorhexidine 0.12% mouth rinse twice daily for 2 weeks. Follow‐up occurred at 3 weeks for suture removal, then at 3 months, 6 months, and annually. A 12‐month interval was selected to allow sufficient periodontal and bone healing while maintaining favorable conditions for secondary transplantation. As part of the prospectively designed staged protocol, secondary autotransplantation was indicated based on criteria established prior to treatment initiation: periodontal probing depths exceeding 5 mm at 12 months, absence of radiographic signs of pulp canal obliteration, evidence of progressive bone loss, or periapical pathology. These criteria were applied consistently across all three cases and formed the basis for the planned transition from first‐stage to definitive transplantation. Patient satisfaction was assessed using a nonvalidated clinical questionnaire evaluating chewing ability, pain/sensitivity, aesthetic satisfaction, oral hygiene maintenance, and overall satisfaction using a 10‐point Likert scale (1 = *very poor*/*severe problems*, 10 = *excellent*/*no problems*). Results should be interpreted descriptively given the nonvalidated instrument and small sample size. All patients tolerated both procedures without requiring protocol modification. Follow‐up compliance was confirmed at all scheduled time points.

### 2.2. Imaging

CBCT scans were acquired using an i‐CAT Next Generation system (KaVo, Germany) with a voxel size of 0.3 mm, tube voltage of 120 kV, current of 5 mA, exposure time of 4 s, and a field of view of 13 × 16 cm [6]. Panoramic radiographs were obtained using Planmeca Viso G7. Serial imaging used standardized institutional positioning protocols.

### 2.3. Patient Data

Data collection included demographics, medical and dental history, family history, and extraoral and intraoral examination findings.

### 2.4. Outcomes

Primary outcomes were periodontal healing (probing depths), pulpal vitality, and functional restoration. Pulp vitality was assessed by combined clinical and radiographic findings: continued root development, absence of symptoms, absence of periapical radiolucency, and pulp chamber obliteration as indicators of vital pulp response. Direct sensibility testing was considered unreliable in teeth with obliterated pulp chambers due to delayed and attenuated responses [14]. Final outcomes were assessed 24 months after second‐stage autotransplantation in all cases (Table 1). Sex was not analyzed as a primary variable because success is driven by biological factors such as root development stage and periodontal ligament viability [8]. At final follow‐up, all transplanted teeth were additionally evaluated using the Digitest 3 Pulp Vitality Tester (Parkell Inc., Edgewood, New York, United States), demonstrating positive responses in all cases.

**Table 1:** Case series assessment of variables.

	Patient case						
	1		2		3		
Sex	Female		Male		Female		
Age of the patient	16	17	18	19	17	18	18
Donor tooth (first transplant)/donor tooth (second transplant)	18	28	48	28	28	38	18
Description	First autotransplantation from opposite side (same jaw)	Second transplantation from the same jaw, same side	First autotransplantation from opposite side (same jaw)	Second autotransplantation from the opposite jaw, same side	First autotransplantation from opposite side (same jaw)	Second autotransplantation from the opposite jaw, opposite side	Straightening a donor′s tooth
Use of a 3D printed replica	No	Yes	No	Yes	No	Yes	No
Development stage of the donor′s tooth, day of surgery (Moorrees)	3 (1/3 root formed)	4 (more than half)	3 (1/3 root formed)	4 (more than half)	4 (more than half)	4 (more than half)	4 (more than half)
Diagnosis of the recipient region	Ectopic	Autotransplanted tooth (survived)	Follicular cyst	Autotransplanted tooth (survived)	Primary failure of eruption	Autotransplanted tooth (survived)	Mesioangular position (ectopia)
Last control after autotransplantation	1 year	2 years	1 year	2 years	1 year	2 years	2 years
Pulp status at last control	—	PCO	—	PCO	—	PCO	PCO
Occlusal plane optimal	No	Yes	No	Yes	Yes	Yes	Yes
Enlarged periodontal pocket	Yes	No	Yes	No	Yes	No	No
Root development stage at final follow‐up (Moorrees)	3 (1/3 root formed)	6 (growth finished)	3 (1/3 root formed)	6 (growth finished)	5 (open apex)	6 (growth finished)	6 (growth finished)
Sign of Ankylosis	No	No	No	No	No	No	No
Sign of resorption	Transient external resorption after first transplant (resolved before second transplant)	No	Transient external resorption after first transplant (resolved before second transplant)	No	Transient external resorption after first transplant (resolved before second transplant)	No	No

Abbreviation: PCO, pulp chamber obliteration.

### 2.5. Donor Tooth Selection

All donor teeth were Moorrees 3–4 development stage. The preferred donor tooth was selected from the same quadrant. If this was not possible, a tooth from the same jaw in another quadrant was used, followed by a tooth from the opposite jaw. All donor teeth were carefully evaluated. Teeth with a complicated anatomy or difficult to extract were not selected. Quadrants containing teeth with a poor prognosis were avoided, especially if those teeth had the potential to erupt into a functional position. In cases with a compromised recipient site, multiple third molars were not extracted at once, and at least one donor tooth was preserved as a reserve for possible future use. For the staged protocol, first‐stage donors were selected at Moorrees stage 3, prioritizing osteogenic potential and periodontal ligament viability over root length, as these teeth were intended to preserve alveolar architecture rather than provide definitive function. Second‐stage donors were selected at Moorrees stage 4, prioritizing root development sufficient for long‐term periodontal stability and functional restoration.

## 3. Case 1: Ankylosed Tooth With Oroantral Communication

### 3.1. Patient Information and Chief Complaint

A healthy 16‐year‐old female was referred for primary eruption failure of Tooth 26. Chief complaints were pain (VAS 4–6/10), masticatory difficulty, and aesthetic concerns. After 24 months of orthodontic extrusion, a 2.5‐cm bone defect was present, with root exposure on Tooth 25 (distal) and Tooth 27 (mesiobuccal). Dental history included 24 months of attempted extrusion of Tooth 26, no history of trauma, and regular preventive care. No relevant family or psychosocial history was reported. Extraoral examination showed no facial asymmetry, temporomandibular joint dysfunction, or palpable lymphadenopathy. Intraoral examination showed good oral hygiene, no active caries, probing depths of 2–3 mm except at the affected site, healthy soft tissues, and adequate attached gingiva.

### 3.2. Clinical and Radiographic Findings

At the affected site, probing depths were 8–12 mm with suppuration. Imaging showed loss of the periodontal ligament space and localized vertical bone loss. Differential diagnoses included ankylosis, severe external root resorption, chronic periodontal disease with secondary eruption failure, and dentoalveolar infection. Ankylosis was diagnosed based on absent periodontal ligament space, a metallic percussion sound, and lack of mobility despite advanced bone loss.

### 3.3. Diagnostic Considerations

CBCT was used to assess sinus involvement and whether sufficient bone would remain for transplantation after extraction.

### 3.4. Therapeutic Interventions

#### 3.4.1. First Autotransplantation (Tooth 18 → Site 26)

The donor tooth was transplanted freehand into Site 26 under local anesthesia with intravenous sedation because of anticipated surgical complexity. 3D planning was not used because the recipient defect exceeded the donor dimensions, allowing flexible positioning. An oroantral communication was identified during extraction of Tooth 26. The dental follicle attached to donor tooth 18 was preserved during extraction and repositioned over the communication to act as a biologic barrier between the maxillary sinus and oral cavity, enabling simultaneous oroantral closure and autotransplantation. Total operative time was 125 min; extra‐alveolar time was 30 s. The transplanted tooth was stabilized in infraocclusion with sutures. At 12 months, probing depths of 5–6 mm around Tooth 18, in the absence of periapical obliteration, indicated a nonrestorable endodontic–periodontal prognosis. The first transplanted tooth was therefore extracted, and a second‐stage autotransplantation was performed using Tooth 28 as the definitive donor, capitalizing on the alveolar architecture preserved by the first‐stage autotransplant.

### 3.5. Adverse Events

Intraoperative oroantral communication was managed as described above, and there were no postoperative complications.

#### 3.5.1. Second Autotransplantation (Tooth 28 → Site 26)

To optimize sinus floor elevation and recipient site morphology, the sinus tapping technique (crestal approach sinus lift) was applied [15]. Tooth 28 was transplanted to Site 26. Operative time was 35 min.

### 3.6. Follow‐Up and Outcomes

**Figure 1:**
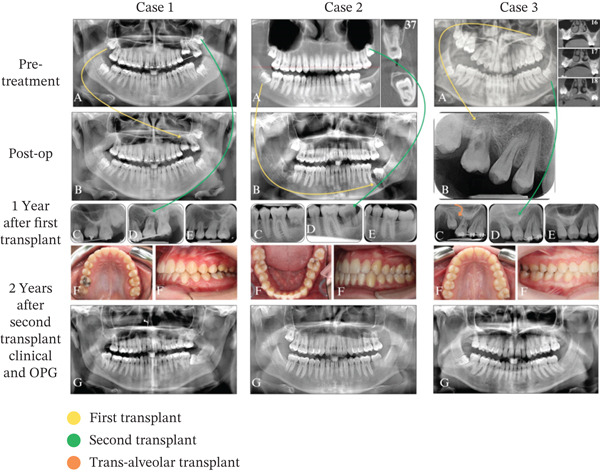
Initial presentation and treatment progression of three patients undergoing two‐stage autotransplantation with 3D planning and orthodontic coordination. (A) Initial panoramic and CBCT radiographs showing root development, recipient site condition, and donor tooth position before treatment. (B) Immediate postoperative radiographs after the first autotransplantation. (C) One‐year follow‐up radiographs after the first autotransplantation. (D) Immediate postoperative radiographs after the second autotransplantation. (E) Two‐year follow‐up radiographs after the second autotransplantation. (F) Intraoral photographs (occlusal and buccal views) demonstrating arch form and esthetic outcome. (G) Final panoramic radiographs at 2 years after the second autotransplantation follow‐up. Colored arrows indicate the sequence and type of autotransplantation procedures: Yellow denotes the first autotransplantation, green denotes the second autotransplantation, and orange denotes transalveolar autotransplantation.

At 24 months, radiographs showed maintained alveolar height and integration of the transplant in the occlusal plane. Probing depths normalized to 2–3 mm. Pain resolved (VAS 0–1/10). The patient′s satisfaction score was 8/10. The patient stated “Initially, I was afraid to chew food on that side, but now I have the feeling that the tooth has grown there naturally” (Figure 1 and Table 1).

## 4. Case 2: Extensive Periodontal Defect With Cystic Pathology

### 4.1. Patient Demographics and History

Patient no. 2 is an 18‐year‐old male with no systemic conditions, regular medication use, or smoking history. He had not received prior dental treatment. Tooth 37 was missing, and CBCT confirmed the absence of a developing third molar (Tooth 38) in the same region. There was no relevant family, psychosocial, or genetic history.

### 4.2. Extraoral Examination

No facial swelling or lymphadenopathy was present.

### 4.3. General Intraoral Findings

Oral hygiene was moderate, with localized plaque accumulation at the affected site. Generalized probing depths were 2–3 mm. No other active caries or signs of periodontal disease were observed. Mucosa was intact except at the affected site.

### 4.4. Clinical and Radiographic Findings

CBCT showed a radiolucent lesion surrounding the crown of the unerupted mandibular left second molar, with corticated borders and associated bone expansion. These features were consistent with a follicular cyst. Because the tooth showed complete root formation and an unfavorable position, transalveolar repositioning was not considered. Surgical enucleation was performed, and histopathological analysis confirmed the diagnosis.

### 4.5. Diagnostic Considerations

Assessment of residual bone volume was limited by the size of the cystic defect. The congenital absence of Tooth 38 required a modified sequential transplantation strategy using a contralateral donor.

### 4.6. Therapeutic Interventions

#### 4.6.1. First Autotransplantation (Tooth 48 → Site 37)

A sequential autotransplantation protocol was planned to allow lesion removal while preserving alveolar bone. Tooth 48 was selected as the initial immature donor tooth. Surgical management included cyst enucleation and defect debridement, followed by recipient site preparation and autotransplantation of Tooth 48. Total operative time was 140 min, with an extra‐alveolar time of 120 s due to the complexity of the lesion and recipient site preparation. At the 12‐month review, clinical and radiographic findings of incomplete integration, progressive periodontal deterioration, and suboptimal donor tooth development indicated a hopeless prognosis for the first transplant. The tooth was extracted and a second staged autotransplantation was performed using Tooth 28 as the definitive donor, utilizing the alveolar volume maintained by the initial transplantation.

#### 4.6.2. Second Autotransplantation (Tooth 28 → Site 37)

A second transplant was performed using Tooth 28 as the definitive donor. Operative time was 50 min, with further refinement of the recipient site.

### 4.7. Adverse Events

No intraoperative or postoperative surgical complications occurred. The first‐stage autotransplant met the prospectively defined criteria for transition to the second stage, as described above. No intraoperative or postoperative surgical complications occurred.

### 4.8. Follow‐Up and Outcomes

No orthodontic treatment was performed. Probing depths normalized to 2–3 mm with radiographic evidence of bone regeneration and stable periodontal architecture. Patient satisfaction score was 9/10. At 24 months, the patient stated “Sometimes I even forget that something was transplanted, because everything is perfect” (Figure 1 and Table 1).

## 5. Case 3: Primary Eruption Failure With Concurrent Orthodontic Intervention

### 5.1. Patient Information and Chief Complaint

A 17‐year‐old female presented with unilateral primary eruption failure affecting Teeth 16 and 17, resulting in a unilateral open bite and speech difficulties.

### 5.2. Patient Demographics and History

She had no systemic conditions or medications. Primary eruption failure was diagnosed at age 14 based on arrested eruption without mechanical obstruction. A prior orthodontic consultation recommended extraction and prosthetic replacement, which was declined. She had no history of trauma and attended regular dental examinations. Family history revealed a maternal aunt with a similar eruption pattern, suggesting a possible familial component. The patient reported concerns about speech and aesthetics. Primary failure of eruption was diagnosed clinically.

### 5.3. Extraoral Examination

The patient exhibits mild facial asymmetry on affected side due to lack of vertical development, with no TMJ symptoms.

### 5.4. General Intraoral Findings

She had excellent oral hygiene, no caries, healthy periodontal tissues with 2–3 mm probing depths on erupted teeth. A 1.5‐cm unilateral posterior open bite was noted on right side, and soft tissue texture was normal.

### 5.5. Clinical and Radiographic Findings

Clinical examination confirmed absence of Teeth 16 and 17 in the occlusal plane and the presence of an open bite on the affected side. CBCT imaging demonstrated impacted posterior maxillary teeth and altered bone architecture.

### 5.6. Differential Diagnosis

Differential diagnoses included primary failure of eruption, mechanical obstruction, ankylosis, regional odontodysplasia, and cleidocranial dysplasia (excluded by normal clavicular development). Primary failure of eruption was confirmed by adequate space, absence of mechanical obstruction on CBCT, failure to respond to orthodontic forces, and familial history.

### 5.7. Diagnostic Challenges

Determining whether affected teeth had any ankylosis that would complicate extraction and assessing the quality of surrounding bone for transplantation recipient site were the diagnostic challenges.

### 5.8. Therapeutic Interventions

#### 5.8.1. First Autotransplantation (Tooth 28 → Site 16)

A combined orthodontic–surgical approach was planned, integrating autotransplantation and tooth uprighting. A freehand autotransplantation was performed. Tooth 28 was transplanted to the site of Tooth 16, with a total operative time of 90 min and an extra‐alveolar time of 180 s. At 1‐year review, periodontal probing depths of 7–8 mm, and a periapical radiolucency indicated a compromised endodontic–periodontal prognosis and resorption of the first transplant. The tooth was assessed as nonrestorable, extracted, and a second staged autotransplantation was performed following alveolar healing and remodeling of surrounding tissues.

#### 5.8.2. Second Autotransplantation and Uprighting (Tooth 38 → Site 16 With Uprighting of Tooth 18)

A combined procedure was undertaken, consisting of autotransplantation of Tooth 38 to the maxillary site and uprighting of Tooth 18 to improve occlusal relationships. Surgical time was 75 min, with an extra‐alveolar time of 60 s for the transplanted tooth.

### 5.9. Adverse Events

No intraoperative or postoperative surgical complications occurred. The first‐stage autotransplant met the prospectively defined criteria for transition to the second stage as described above. These findings are consistent with the anticipated biological limitations of immature donors in severely compromised sites and informed the planned second‐stage intervention. No intraoperative or postoperative surgical complications occurred.

### 5.10. Follow‐Up and Outcomes

At 2‐year follow‐up, no ankylosis or pathological root resorption was observed radiographically. The occlusal plane was fully restored, and the unilateral open bite had been corrected. Patient satisfaction score was 10/10. The patient indicated no awareness of the transplant during daily function (Figure 1 and Table 1).

## 6. Results

All three patients completed the sequential double autotransplantation protocol. Table 1 summarizes clinical variables and outcomes. At final follow‐up (24 months after second autotransplantation), all transplanted teeth demonstrated pulp chamber obliteration without clinical symptoms or periapical pathology, interpreted as a vital pulp response, normalized periodontal probing depths (2–3 mm), absence of ankylosis, and optimal positioning within the occlusal plane. No bleeding on probing was observed. No cases required endodontic intervention based on predefined clinical and radiographic criteria. The first‐stage autotransplants did not achieve standalone long‐term success, meeting the predefined criteria for extraction and second‐stage transplantation at 12 months in all cases.

**Figure 2:**
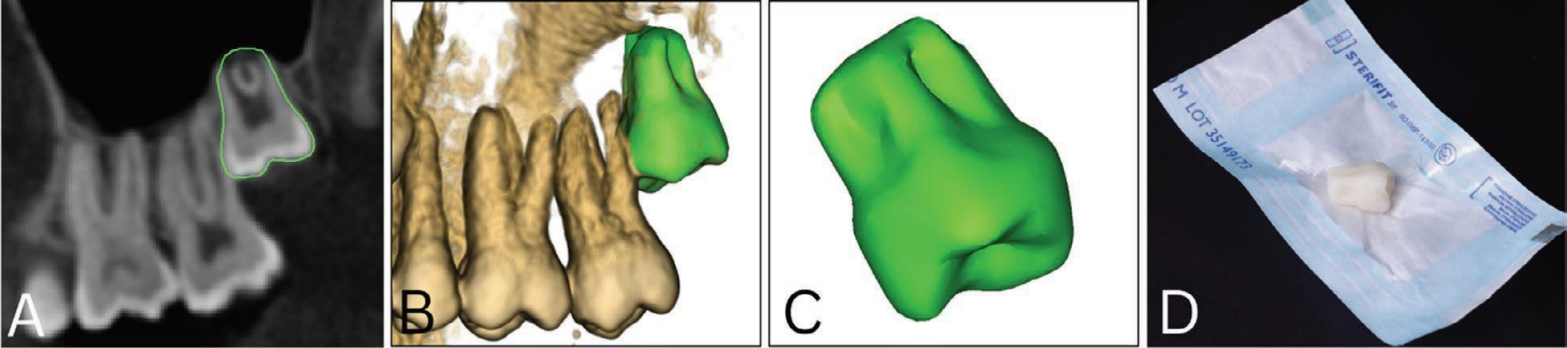
CBCT‐based digital planning and fabrication of a sterilized 3D tooth replica from Case 2. (A) CBCT showing an impacted tooth outlined in green. (B) 3D reconstruction of the jaw with the impacted tooth highlighted in green. (C) Digital 3D model of the tooth. (D) 3D‐printed replica of the tooth on sterile packaging, ready for surgical planning. The replica was sterilized at 121°C in a two‐atmosphere steam sterilization cycle (60 min).

All patients reported normal masticatory function. Patient satisfaction scores ranged from 8/10 (Case 1) to 10/10 (Case 3), with Case 2 reporting 9/10. Figure 1 illustrates treatment progression for all cases. Figure 2 demonstrates the 3D planning and replica printing process.

## 7. Discussion

This report describes a prospectively designed staged autotransplantation protocol developed from broader clinical experience with 146 immature tooth autotransplantations between 2019 and 2025 [13]. In cases with severely compromised recipient sites, conventional single‐stage outcomes were suboptimal, with the recipient site identified as the primary determinant of outcome. The staged protocol was therefore prospectively designed to address this limitation: The first‐stage autotransplant was intended to preserve alveolar architecture and stimulate bone regeneration in advance of definitive transplantation, while simultaneously ensuring sufficient bone volume for implant‐based rehabilitation should skeletal growth preclude earlier definitive treatment. These patients represented complex situations in which the recipient site, not the donor tooth, was the primary determinant of outcome.

The staged protocol increases financial costs through repeated surgical procedures, CBCT imaging, digital planning, and prolonged follow‐up. However, biological cost remains relatively low as the approach uses autologous immature third molars that would otherwise be extracted without functional use. In growing patients with severely compromised sites, alternatives such as guided bone regeneration, titanium mesh reconstruction, and implant therapy may involve substantially greater biological and financial costs and may be contraindicated due to ongoing skeletal growth [2, 16, 17]. Sequential autotransplantation therefore represents a biologically conservative strategy preserving periodontal ligament function and alveolar development. The present cases suggest that recipient site condition is a more critical determinant of long‐term success than donor tooth developmental stage alone in severely compromised situations. Although donor teeth were selected at favorable Moorrees developmental stages, all recipient sites demonstrated substantial biological compromise including extensive bone defects, chronic periodontal destruction, cystic pathology, oroantral communication, or altered eruption patterns. Despite improved surgical precision through CBCT‐guided planning and 3D‐printed replicas [6, 13], technical accuracy alone may not overcome severe biological limitations of compromised recipient sites.

Autotransplantation demonstrates survival rates of 80.0%–97.5% with appropriate case selection [4, 18]. In the present case series, the first‐stage autotransplant contributed to preservation of alveolar architecture and periodontal space during healing. A recent in vitro study reported regenerative effects from periodontal ligament‐derived cells following autotransplantation [11], though the exact mechanism remains under investigation. Pulp chamber obliteration occurred in all successfully transplanted teeth. This tertiary dentin deposition reflects vital pulp tissue responding to transplantation trauma rather than dystrophic calcification, as evidenced by the absence of periapical pathology and continued root development [19]. In the patient involving a large cystic defect, this staged approach allowed preservation and subsequent remodeling of the alveolar bone, consistent with recent reports on autotransplantation in cyst‐associated defects [20]. Transient external root resorption after first transplantation represents a limited inflammatory response to surgical injury in a compromised recipient site with reduced bony housing and vascular supply [21]. In immature donor teeth, impaired revascularization may lead to pulp necrosis, sustaining inflammatory resorption through bacterial diffusion from the necrotic pulp [22]. Progressive alveolar healing and remodeling subsequently re‐establish vascularized bone and periodontal support, creating more favorable conditions for secondary transplantation [21].

Integration of CBCT planning and 3D‐printed templates reduces extra‐alveolar time and increases surgical accuracy [7, 13], while minimizing Hertwig epithelial root sheath injury [5]. The root development stage represents the most critical predictor of transplant success, with teeth at Moorrees′ stages 2–4 demonstrating superior pulp survival compared with mature teeth [23, 24]. This staged approach maintains periodontal space and stimulates alveolar development, creating conditions for subsequent definitive transplantation. Furthermore, this intervention may offer an alternative to space maintainers, which carry caries and periodontal risks [25], and to premature implant placement, contraindicated in growing patients [2, 16, 17, 26]. The protocol increases treatment complexity and requires surgical expertise and appropriate equipment. Additional surgical procedures can impair root development in early stages [23], necessitating timing considerations. Several important limitations must be acknowledged. First, the sample size of three cases does not support a protocol recommendation and limits statistical interpretation. Second, the staged protocol was applied in cases with exceptionally compromised recipient sites prospectively identified as unsuitable for conventional single‐stage autotransplantation; generalizability to less compromised sites should not be assumed. Third, first‐stage donors in Cases 1 and 2 were deliberately selected at Moorrees stage 3, knowing their limited root development made long‐term standalone success unlikely, as the intent was osteogenic space preservation rather than definitive replacement. Fourth, 3D‐printed replicas were used for second‐stage but not first‐stage transplantations, introducing a potential confound in attributing differential outcomes to the staged approach versus improved surgical precision at the second stage. Fifth, lack of ethnic diversity limits generalizability, although the biological principles of autotransplantation are expected to apply across populations.

In three cases where first‐stage immature autotransplants did not achieve standalone success in severely compromised recipient sites, second‐stage transplantation with more mature donors and CBCT‐guided planning achieved favorable outcomes at 24‐month follow‐up. Whether the first‐stage autotransplant actively improved conditions for the second stage, or whether the second stage would have succeeded regardless, cannot be determined from this case series alone. Recipient site condition, rather than donor tooth development alone, appears to be the primary determinant of outcome in such cases. Validation through larger prospective controlled studies is required before this staged approach can be recommended as a clinical protocol.

## Author Contributions

M.L., J.E.O., and S.E.U. contributed to the conception and design of the case series. M.L., A.A., and G.S. acquired the clinical data. M.L. and S.E.U. drafted the manuscript. M.L., S.E.U., A.A., G.S., M‐T.F., and J.E.O. critically revised the manuscript for important intellectual content. M.L. and G.S. performed the surgical procedures. M‐T.F. and J.E.O. contributed to the pediatric clinical management. A.A. contributed to orthodontic assessment and treatment planning. S.E.U. performed the imaging analyses.

## Funding

M.L. and S.E.U. acknowledge financial support from the European Union′s Horizon 2020 research and innovation programme under Grant Agreement No. 857287 for the Baltic Biomaterials Centre of Excellence (BBCE). S.E.U. also acknowledges support as principal investigator of the Scientist Grant “EpiDentLatvia” (RSU‐ZG‐2024/1‐0044), conducted as part of the project “RSU Internal and RSU with LASE External Consolidation” (Project No. 5.2.1.1.i.0/2/24/I/CFLA/005), funded by the European Union Recovery and Resilience Facility and the state budget of the Republic of Latvia.

## Disclosure

The authors assume full responsibility for accuracy and integrity. All authors approved the final version of the manuscript and agreed to be accountable for all aspects of the work.

## Ethics Statement

Ethical approval was obtained from the Rīga Stradiņš University Research Ethics Committee (No. 6‐1/08/12, July 23, 2020; 2_PĒK‐4/530/2026, April 16, 2026). Written informed consent was obtained from all patients. Consent included explicit authorization for publication of clinical photographs, radiographic images, and case details. Copies of written consent are available for review upon request by the journal editor.

## Conflicts of Interest

The authors declare no conflicts of interest.

## Data Availability

The data supporting the findings of this study are available within the article and its supporting information. Individual patient data cannot be shared publicly due to privacy and consent restrictions.
